# A report on the development of COVID-19 guidelines for rehabilitation professionals in African settings

**DOI:** 10.11604/pamj.2021.38.129.26311

**Published:** 2021-02-04

**Authors:** Etienne Ngeh Ngeh, Nnenna Nina Chigbo, Zillah Whitehouse, Emelie Moris Anekwu, Lela Mukaruzima, Lungile Mtsetfwa, Rogers Kitur, Mary Wetani Agoriwo, Priscillah Ondoga, Maurice Douryang, Lynn Cockburn

**Affiliations:** 1 African Rehabilitation Network (AFRENET),; 2 Research Organization for Health Education and Rehabilitation-Cameroon (ROHER-CAM),; 3 Physiotherapy Department, Regional Hospital Bamenda, Bamenda, North West Region, Cameroon,; 4 Department of Physiotherapy, University of Nigeria Teaching Hospital Enugu, Enugu, Nigeria,; 5 Department of Physiotherapy, Faculty of Medicine, Mbarara Unversity of Science and Technology, Mbarara, Uganda,; 6 Department of Physiotherapy, Alex Ekwueme Federal University Teaching Hospital, Ebonyi State, Nigeria,; 7 LnV Physiotherapy Service, Mbabane, Eswatini,; 8 Kapsabet County Referral Hospital, Emgwen, Kenya,; 9 Department of Physiotherapy and Rehabilitation Science, University of Health and Allied Sciences/Ho Teaching Hospital, Ho, Ghana,; 10 Rehabilitation Unit, Faculty of Medicine and Pharmaceutical Sciences, University of Dschang, Dschang, Cameroon,; 11 Department of Occupational Science and Occupational Therapy, University of Toronto, Toronto, Canada

**Keywords:** COVID-19, guidelines, rehabilitation, Africa

## Abstract

COVID-19 is the disease caused by SARS-CoV-2, one of a large family of coronaviruses. Severe forms of the disease can lead to respiratory failure with multiple organ failure necessitating rehabilitation in both acute and long-term care. With the increasing prevalence of COVID-19 and rehabilitation needs, the African Rehabilitation Network (AFRENET) produced a guidance document to assist in reducing variation in clinical practice among rehabilitation professionals in the Africa Region. This report outlines the process of the guideline development.

## Introduction

COVID-19 is the disease caused by SARS-CoV-2, one of a large family of coronaviruses [1]. Common symptoms include fever, dry cough, sore throat, headache, generalized weakness or fatigue, and difficulty breathing. Severe forms of the disease can lead to respiratory failure with multiple organ failure necessitating rehabilitation in both acute and long-term care [1]. The African region has not been spared and is faced with similar rehabilitation challenges as other parts of the world. The rehabilitation needs of COVID-19 survivors are diverse including physical, cognitive, and psychosocial issues necessitating the services of different rehabilitation professionals [1, 2]. At the time of writing this article, more than 50% of all countries in Africa were experiencing community transmission with increasing numbers of patients with the severe forms of the disease, and rising case fatality rates from the disease [3].

In Africa, COVID-19 is reported through the WHO AFRO (World Health Organization Africa Region Office), and the WHO EMRO (Eastern Mediterranean Regional Office). The first cases of COVID-19 were confirmed on 14 February 2020 in Egypt, and soon after in Algeria, Nigeria, and Senegal [4]. Since then the numbers of countries reporting cases, active cases with moderate to severe form of the disease, and deaths have been rising steeply [3].

Although African health systems are growing, there are still many places where health services are inadequate. The COVID-19 pandemic has increased the strain on already fragile health systems with insufficient rehabilitation services across the region also challenged. Many existing rehabilitation services across Africa often suffer from lack of infrastructure and human resources with increased workload for rehabilitation professionals. There are few clinical practice guidelines in rehabilitation that were adapted or contextualized to the region to improve practice. To the best of our knowledge there were no existing guidelines developed, adapted, or contextualized for rehabilitation professionals during this period of COVID-19.

## Methods

As the pandemic grew, rehabilitation professionals took notice of the increasing rehabilitation needs in the region. The African Rehabilitation Network (AFRENET) was formed by a diverse group of rehabilitation professionals in Africa. We thought of innovative ways to mobilize resources and deliver effective services despite the extra burden imposed by COVID-19. With these initiatives and efforts, we created a community of practice involving rehabilitation professionals practicing in Africa, using WhatsApp and Zoom to bring practitioners together from many countries to discuss the important role of rehabilitation in the region during the crisis.

One action taken by AFRENET was to develop a set of guidelines for rehabilitation practitioners to improve on the effectiveness of service delivery, decrease costs, prevent avoidable mistakes, and reduce variation in clinical practice in the region. This process was inspired by, and adapted with permission from, a group at McMaster University in Canada who had already published rehabilitation guidelines during the COVID-19 pandemic [5], and another guideline published in the Journal of Physiotherapy by Thomas and colleagues [6]. To avoid duplication of efforts, we adapted recommendations based on best available evidence and contextualized and expanded them by combining them with findings and experiences of frontline rehabilitation professionals from the Africa region on COVID-19. The group worked together to create the guideline through 1) reviewing the literature, 2) creating an online document for asynchronous dialogue; and 3) consulting with experts.

**Reviewing the literature:** given the fact that COVID-19 is a new disease of pandemic proportions and data is still evolving, very little was published in the academic literature specifically about COVID-19 and rehabilitation. At that time, we found no academic literature specific to COVID-19 and rehabilitation emanating from the Africa Region. As such we gathered literature that was being shared from professional networks, gray literature, and from academic databases on related topics across the globe. We also identified and adopted guidelines from other settings within the region. All the available data was collected into a Zotero library and made accessible to all members of the rehabilitation professional group.

**Online discussions:** following the group initiative, a draft list of guidelines was developed based on existing literature. A Google document was generated with the drafted guidance document to allow for contributions. There was significant discussion which recognized the diversity, the strengths, and the challenges across the continent. Discussion centered on what was feasible and acceptable within the African context based on existing infrastructure and human resources. Contributors talked about the existence of a very low ratio of health professionals to patients and need to improve practice and quality of care during this period. This lack of health personnel is even more evident among rehabilitation workers such as community-based rehabilitation workers (CBRWs), occupational therapists (OTs), physiotherapists (PTs), speech and language therapists (SLTs) in the continent. Other frontline workers who had firsthand experience or who had participated in developing any form of guidance document related to rehabilitation at the institutional or national level also shared their experiences for discussion on what was most relevant and practical in the region.

The authors recognized that the situations in various settings, cities, regions, and countries can be very different based on infrastructure and availability of rehabilitation professionals. We discussed the need for guidelines to be applicable in a variety of settings. For example, there are significant differences between the management of COVID-19 in the better resourced healthcare settings, and the many other African settings where rehabilitation is not fully supported. Social and cultural conditions were also considered where rehabilitation professionals represented and shared their experiences. It was agreed that the document would not replace institutional policies and practices but rather supplement existing efforts and resources put in place to improve client/patient and healthcare professionals´ safety and practice during this period.

**Consultation with experts:** the complete draft guideline for rehabilitation professionals in Africa was then forwarded to experts in guideline development and specialist clinicians in rehabilitation including the team at McMaster University, Ontario, Canada and the Community-based rehabilitation Africa Network, who provided detailed constructive feedback. This feedback was then discussed among the writing group members and integrated into the document.

## Results

The result was a set of guidelines called *Rehabilitation of patients with COVID-19 in African Settings: Guidance for Community Based Rehabilitation Workers, Physiotherapists, Occupational Therapists, Speech and Language Therapists, and Assistant*s. The guidelines are available online. Updates of this document will also be posted on these sites when made. The final guidance document is divided into two main sections. The first section is composed of seven interconnected key considerations applicable to all rehabilitation providers. Each of these key considerations is further supported with specific recommendations. The key considerations are: i) assessment of risk and actions to be taken to reduce risk and spread of COVID-19 in rehabilitation settings; ii) working as a team; iii) avoidance of person to person contact as much as possible; iv) anticipation of an increased demand of workforce; v) type of personal protective equipment (PPE) needed for patient contact; vi) inclusion of people with other impairments and disabilities apart from those due to COVID-19; vii) streamlining of documentation procedures.

The second section is composed of best practice statements for specific rehabilitation providers notably, statements for community-based rehabilitation workers for homes and communities where the present COVID-19 patients are established and where the presence of COVID-19 is unknown. We also provided specific statements for different phases of the rehabilitation process in hospital settings, as well as therapeutic procedures for physiotherapists, occupational therapists, and speech and language therapists.

## Discussion

The guidance document was designed to be practical and to meet the need of a diverse group of rehabilitation professionals (CBRWs, PTs, OTs and SLTs) in the African context. While clinical practice guidelines have evolved from being opinion-based to evidence-informed with an increasing sophisticated methodology in development [7], experts in development, implementation, and evaluation of clinical practice guidelines advocate for making recommendations available to improve practice even when the evidence is considered insufficient [8]. With this background and other context specific considerations, we developed the guidance document for rehabilitation providers in Africa settings. These guidelines are intended to be used in complement with other resources from credible sources such as the WHO, national Ministries of Health, and guidelines from professional associations and world professional bodies.

**Challenges:** from the conception to realization of the guidelines, there were challenges principally due to insufficient availability of rehabilitation professionals with skills on guideline development and COVID-19 disease. This perceived lack of skilled professionals was further compounded by the absence of a well-established international or national guideline development group or manual in the region such as the Scottish Intercollegiate Guideline Network (SIGN) [9] or the Australian National Health and Medical Research Council (NHMRC) [10]. Other factors included lack of funding, poor internet availability, and frequent interrupted power supply.

## Conclusion

We report a successful adaptation and contextualization of a clinical practice guideline for rehabilitation professionals working in African settings with patients with or without COVID-19, through the efforts of a small team of dedicated volunteers with variable understanding of developing clinical practice statements and recommendations. We encourage similar efforts and collaboration in rehabilitation within the region.

## References

[1] Pan American Health Organization (PAHO) World Health Organization (WHO). Rehabilitation considerations during the COVID-19 outbreak.

[2] Sheehy Larry Mary (2020). Considerations for Postacute Rehabilitation for Survivors of COVID-19. JMIR Public Health Surveill.

[3] World Health Organization Regional Office for Africa Africa records over 200 000 COVID-19 cases.

[4] World Health Organization Regional Office for Africa Coronavirus (COVID-19).

[5] McMaster University's School of Rehabilitation Science Patient Safety Learning Team. Rehabilitation for patients with COVID-19: guidance for Occupational Therapists, Physical Therapists, Speech-Language Pathologists and Assistants (April 2020).

[6] Thomas P, Baldwin C, Bissett B, Boden I, Gosselink R, Granger CL (2020). Physiotherapy management for COVID-19 in the acute hospital setting: clinical practice recommendations. J Physiother.

[7] Kredo Tamara, Bernhardsson Susanne, Machingaidze Shinghai, Young Taryn, Louw Quinette, Ochodo Eleanor (2016). Guide to clinical practice guidelines: the current state of play. Int J Qual Health Care.

[8] Neumann Ignacio, Schünemann Holger J (2020). Guideline groups should make recommendations even if the evidence is considered insufficient. Can Med Assoc J.

[9] Scottish Intercollegiate Guidelines Network SIGN50: A guideline developer’s handbook.

[10] National Health and Medical Research Council A Guide to the development, implementation and evaluation of clinical practice guidelines.

